# Montelukast: More than a Cysteinyl Leukotriene Receptor Antagonist?

**DOI:** 10.1100/tsw.2010.229

**Published:** 2010-12-14

**Authors:** Gregory R. Tintinger, Charles Feldman, Annette J. Theron, Ronald Anderson

**Affiliations:** ^1^ Department of Internal Medicine, Faculty of Health Sciences, University of Pretoria, South Africa; ^2^ Department of Internal Medicine, Faculty of Health Sciences, University of the Witwatersrand, Johannesburg, South Africa; ^3^ Medical Research Council Unit for Inflammation and Immunity, Department of Immunology, University of Pretoria, and Tshwane Academic Division of the National Health Laboratory Service, Pretoria, South Africa

**Keywords:** chronic obstructive pulmonary disease, cyclic AMP, cysteinyl leukotrienes, cystic fibrosis, histone acetyltransferase, 5-lipoxygenase, cyclic nucleotide phospodiesterase, sepsis, viral bronchiolitis

## Abstract

The prototype cysteinyl leukotriene receptor antagonist, montelukast, is generally considered to have a niche application in the therapy of exercise- and aspirin-induced asthma. It is also used as add-on therapy in patients whose asthma is poorly controlled with inhaled corticosteroid monotherapy, or with the combination of a long-acting β(2)-agonist and an inhaled corticosteroid. Recently, however, montelukast has been reported to possess secondary anti-inflammatory properties, apparently unrelated to conventional antagonism of cysteinyl leukotriene receptors. These novel activities enable montelukast to target eosinophils, monocytes, and, in particular, the corticosteroid-insensitive neutrophil, suggesting that this agent may have a broader spectrum of anti-inflammatory activities than originally thought. If so, montelukast is potentially useful in the chemotherapy of intermittent asthma, chronic obstructive pulmonary disease, cystic fibrosis, and viral bronchiolitis, which, to a large extent, involve airway epithelial cell/neutrophil interactions. The primary objective of this mini-review is to present evidence for the cysteinyl leukotrien–independent mechanisms of action of montelukast and their potential clinical relevance.

## INTRODUCTION

Montelukast is a prototype, selective, pharmacological antagonist of type 1 cysteinyl leukotriene receptors ($CysLT_1Rs$). These G protein–coupled receptors recognize the CysLTs $LTD_4$ and $LTC_4$/$LTE_4$ expressed on the plasma membrane of structural (epithelial, fibroblasts/myoblasts, smooth muscle) and inflammatory cells, including neutrophils, monocytes/macrophages, mast cells, basophils, dendritic cells, and lymphocytes[1]. Following their interaction with $CysLT_1Rs$ on target cells, CysLTs recruit and activate inflammatory cells; enhance vascular permeability and bronchial hyper-reactivity; promote airway remodeling, which is consequent to the release of proteolytic enzymes such as elastase and matrix metalloproteinases (MMPs); and prime/sensitize neutrophils for hyper-reactivity on subsequent exposure to formyl peptides[1,2,3,4], leading to enhanced release of elastase and MMP-8[4].

Montelukast effectively antagonizes the proasthmatic/proinflammatory/priming activities of CysLTs and forms part of numerous international guidelines for asthma therapy[5]. Interestingly, recent evidence suggests that montelukast possesses a range of secondary anti-inflammatory activities, apparently unrelated to antagonism of $CysLT_1Rs$. These include inhibition of the enzymes 5-lipoxygenase[6], histone acetyltransferase (HAT)[7], and adenosine 3',5'-cyclic monophosphate (cAMP) phosphodiesterase[8], as well as interference with purinergic P2Y receptors[6], and inhibition of eosinophil adhesion to vascular endothelium and migration[9]. These $CysLT_1R$-independent, anti-inflammatory mechanisms of action of montelukast may be particularly effective in controlling the corticosteroid-insensitive neutrophil and are the major focus of the current review. Of necessity, however, an overview of these is preceded by a consideration of the role of cAMP in controlling neutrophil-mediated inflammation.

## ANTI-INFLAMMATORY ACTIVITY OF cAMP-ELEVATING AGENTS

cAMP possesses broad spectrum anti-inflammatory activity and has been described as the "master regulator of innate immune cell function"[10]. As a consequence of activation of cAMP-dependent protein kinase A (PKA), cAMP promotes restoration of $Ca^{2+}$ homeostasis in neutrophils and other cell types by multiple mechanisms, including phosphorylative inactivation of phospholipase Cβ3[11], inactivation of store-operated $Ca^{2+}$ channels[12], inhibition of p38 MAP kinase with consequent interference with activation of 5-lipoxygenase[13], and attenuation of an autocrine, leukotriene $B_4$ ($LTB_4$)–mediated secondary wave of $Ca^{2+}$ influx[14].

Elevated cytosolic $Ca^{2+}$ concentrations precede and are a prerequisite for neutrophil degranulation, oxidant production, and release of lipid mediators consequent to activation with chemoattractants, such as platelet-activating factor, formyl peptides, C5a, and $LTB_4$[15]. Following release from cytosolic vesicles, $Ca^{2+}$ is rapidly resequestered into calciosomes by endomembrane ATPases up-regulated by PKA[16]. Intracellular cAMP concentrations are dependent on the activity of adenylate cyclase enzymes, which generate cAMP from ADP and on the activity of intracellular phosphodiesterases (PDEs), which hydrolyze cAMP[17]. Inhibition of PDEs markedly delays cAMP removal from the cytosol, promoting the PKA-mediated clearance of cytosolic $Ca^{2+}$. This accelerated uptake of $Ca^{2+}$ into intracellular stores is associated with significant attenuation of multiple $Ca^{2+}$-dependent pathways, which generate proinflammatory mediators[18,19].

cAMP-elevating agents effectively target multiple proinflammatory pathways[10], but to date have enjoyed limited utility in clinical practice consequent to a narrow therapeutic window (theophylline) or significant adverse effects (theophylline and roflimulast).

## CYSLT$_1$R-INDEPENDENT ANTI-INFLAMMATORY ACTIVITIES OF MONTELUKAST

### 5-Lipoxygenase

Receptor-mediated stimulation of cells of the innate immune system results in $Ca^{2+}$ mobilization and activation of 5-lipoxygenase. The primary consequence is production of the potent neutrophil chemoattractant, $LTB_4$, as well as production of CysLTs by basophils, eosinophils, mast cells, and, to a lesser extent, monocytes/macrophages, all of which possess the necessary enzymes for conversion of $LTA_4$ to CysLTs[1]. Montelukast has been reported to inhibit 5-lipoxygenase in both activated neutrophils and monocytes/macrophages by a mechanism that has not been fully characterized, but which appears to be distinct from antagonism of $CysLT_1Rs$[6,8,13]. Although the concentrations (≥1 μM) of montelukast required to cause significant inhibition of 5-lipoxygenase are higher than those required for complete blockade of $CysLT_1Rs$, they are nevertheless close to peak serum concentrations detected during chemotherapy with this agent[20,21]. Inhibition of the synthesis of CysLTs clearly has the potential to complement montelukast-mediated antagonism of $CysLT_1Rs$, while attenuation of production of $LTB_4$ represents an additional therapeutic activity that may contribute to the control of corticosteroid-insensitive neutrophil-mediated inflammation[22,23]. Recently, however, Steib et al. failed to detect inhibitory effects of montelukast at a fixed concentration of 1 μM (the threshold for inhibition of 5-lipoxygenase) on the production of CysLTs by isolated rat Kuppfer cells activated with zymosan or lipopolysaccharide[24]. This may reflect the relative insensitivity of these cells to montelukast, necessitating higher concentrations of this agent and/or the type of stimulant used.

## Histone Acetyltransferase

Pranlukast, another $CysLT_1R$ antagonist, has been reported to inhibit the activation of the transcription factor, nuclear factor (NF)-κB, in allergen-activated human monocytes, or lipopolysaccharide- or tumor necrosis factor (TNF)–stimulated monocyte/macrophage cell lines, as well as in epithelial and endothelial cell lines by a $CysLT_1R$-independent mechanism[25,26,27,28]. This, in turn, results in decreased transcription of genes encoding proinflammatory proteins, particularly cytokines/chemokines, such as interleukin (IL)-8 and TNF. These inhibitory effects of pranlukast on the activation of NF-κB were apparently achieved by $CysLT_1R$-independent mechanisms because (1) similar effects were observed using a $CysLT_1R$-nonexpressing T-cell line[25], (2) $LTD_4$ did not activate NF-κB in the epithelial or endothelial cell lines[26,27,28], and (3) treatment with the 5-lipoxygenase inhibitor, zileuton, did not attenuate activation of NF-κB in a stressed endothelial cell line[28]. Montelukast has also been reported to inhibit the activation of NF-κB in a human monocyte/macrophage cell line[29]. Other than the relatively high concentrations of montelukast required to achieve this effect, the mechanism of inhibition was not convincingly demonstrated to be independent of $CysLT_1Rs$.

More recently, Tahan et al. reported that montelukast at concentrations of 0.01–10 μM caused significant, dose-related suppression (maximal at 0.1 μM) of IL-8 gene transcription and protein synthesis following activation of a monocyte/macrophage cell line with TNF[7]. Although activation of NF-κBp65, measured by DNA binding, was unaffected, treatment of the cells with montelukast resulted in substantial suppression of HAT activity[7]. The precise molecular mechanism underpinning these inhibitory effects of montelukast on HAT was not established, but may involve interference with the activation of transcriptional coactivator proteins, a prerequisite for histone acetylation, chromatin unwinding, and gene transcription.

## Eosinophil Adhesion

Montelukast has been reported to inhibit eosinophil adhesion by several $CysLT_1R$-dependent mechanisms. These include interference with CysLT-mediated (1) adhesion to intercellular adhesion molecule-1 (ICAM-1) by blocking both the avidity and focal clustering of the β(2)-integrin, CD11b/CD18; and (2) STAT-1–induced up-regulation of ICAM-1 expression on bronchial epithelial cells[30,31]. In addition to these anti-inflammatory mechanisms, montelukast has also been reported to target eosinophil adhesion and migration by $CysLT_1R$-independent mechanisms. These include (1) interference with the interaction of the β(1)-integrin, α4β1, with its counter-receptor, vascular cell adhesion molecule-1[9]; and (2) decreased migration activated by the chemoattractant, 5-oxo-6,8,11,14-eicosatetraenoic acid[32]. While the former activity was evident at 0.1 μM and unaffected by inclusion of the 5-lipoxygenase–activating protein inhibitor, MK886, compatible with a $CysLT_1R$-independent mechanism, the latter effect was observed using 10 μM of this agent and was associated with decreased expression of the urokinase plasminogen receptor and secretion of MMP-9, both of which are required for tissue extracellular matrix digestion[32].

## P2Y Receptors

These are a family of G protein–coupled, purinergic receptors activated by nucleotides, such as ATP, ADP, UTP, and UDP. Nucleotide-mediated activation of these receptors amplifies the reactivity of immune and inflammatory cells, potentiating inflammatory responses[33]. P2Y receptors are expressed by both monocytes/macrophages and neutrophils, with ATP-mediated autocrine stimulation of $P2Y_2$ receptors being intimately involved in neutrophil activation[34,35], while UDP signals via $P2Y_6$ receptors on monocytes/macrophages[13,36].

Although their relative contributions to harmful inflammatory responses remain to be established, P2Y receptors represent potential targets for pharmacological control of inflammation. In this context, it is noteworthy that Mamedova et al.[36] and, more recently, Woszczek et al.[13], reported that montelukast, as well as pranlukast[36] and zafirlukast[13], albeit at micromolar concentrations, antagonize the effects of nucleotides acting at P2Y receptors on both a monocyte/macrophage cell line and primary human monocytes. These effects of montelukast, pranlukast, and zafirlukast were independent of $CysLT_1R$ antagonism and were characterized by inhibition of phospholipase C, resulting in failure to generate inositol triphosphate and mobilize $Ca^{2+}$ from intracellular stores, with consequent decreased production of IL-8[13,36]. The molecular mechanism underpinning the effects of montelukast and the other $CysLT_1R$ antagonists on P2Y receptor–mediated signaling remains to be established.

## Cyclic Nucleotide Phosphodiesterases

We have recently reported that montelukast at concentrations ≥0.5 μM caused dose-related inhibition of the chemoattractant-activated proinflammatory activities of isolated human neutrophils. These included the generation of reactive oxygen species, release of the primary granule protease, elastase, and production of $LTB_4$[8,30]. Montelukast markedly attenuated $LTB_4$ production by PAF-activated neutrophils with maximal inhibition (89%) observed at concentrations of 2 μM[8]. Similar effects have been observed on the generation of CysLTs by primary monocytes treated with montelukast and zafirlukast[13]. The inhibitory effects of montelukast on activated neutrophils appeared to be independent of $CysLT_1Rs$, but were, however, associated with increased levels of cAMP and suppression of the chemoattractant-activated increase in cytosolic $Ca^{2+}$. Montelukast-mediated increases in neutrophil cAMP are likely to underpin the effects of this agent in suppressing the increases in cytosolic $Ca^{2+}$[8,37].

Target identification studies revealed nonspecific cyclic nucleotide PDE inhibitory activity to be the probable mechanism of the cAMP-elevating activity of montelukast[8]. PDE4-subtype-B2 is the predominant PDE in human neutrophils[38] and these cells are extremely sensitive to the inhibitory effects of both selective and nonselective PDE inhibitors such as rolipram[39] and pentoxyphylline[40], respectively. Although on a molar concentration basis it is 10–100-fold less potent than rolipram, it is noteworthy that the inhibitory effects of montelukast on neutrophils are not only substantial, but are also evident at therapeutically/close-to-therapeutically relevant concentrations of this drug[8].

Although unproven, it is possible that the cAMP-elevating activity of montelukast may also underpin the inhibitory effects of montelukast on 5-lipoxygenase, HAT, and eosinophil adhesion mentioned above. Activation of 5-lipoxygenase results in translocation of the enzyme from the cytosol to the nuclear membrane, where it associates with 5-lipoxygenase–activating protein, a process requiring $Ca^{2+}$ and activation of p38 MAP kinase, both of which are counteracted by cAMP[41]. In the case of NF-κB, PKA has been reported to interfere with NF-κB–mediated gene transcription in both monocytes and endothelial cells without affecting DNA binding[42], similar to the effects of montelukast on TNF-activated monocytes/macrophages[7]. Activation of PKA results in phosphorylation of cAMP response elementbinding protein (CREB), which, in turn, competes with the p65 component of NF-κB for limiting amounts of the transcriptional coactivator with intrinsic HAT activity, CREB-binding protein[42]. Furthermore, activation of β-integrins, which is a prerequisite for the firm binding of eosinophils to vascular endothelium, is a $Ca^{2+}$-dependent process, as is the release of MMP-9, which is required for dissolution of extracellular matrices[32].

### Other Agents that Combine Antagonism of CysLTRs and Nonspecific PDE Inhibitory Activity

Secondary, nonspecific PDE inhibitory activity has also been described for other $CysLT_1R$ antagonists, including several of the early, experimental $CysLT_1R$ antagonists, such as FPL55712 and LY171883[43,44]. More recently, CR3465, a novel $CysLT_1R$ antagonist, was reported to possess nonspecific PDE inhibitory activity[45]. In the case of FPL55712 and LY171883, PDE inhibitory activity appeared to represent a limitation in respect of specificity of pharmacological mode of action[43,44]. In the case of CR3465, however, the combination of $CysLT_1R$ antagonism and PDE inhibitory activity was considered to be beneficial because the latter property may confer additional protection by targeting spasmogenic and inflammatory mediators other than CysLTs[45]. From a molecular structure/function perspective, montelukast and CR3465 both possess a quinoline moiety, which may underpin the PDE inhibitory activities of these agents[45,46]. Currently, CR3465 is being developed by Rottapharm/Madaus and has completed preclinical evaluation to enter Phase I clinical trials[47].

Ibudilast, also known as KC-404, AV-411, and MN-166, is an anti-inflammatory agent that also combines CysLTR antagonism and PDE inhibitory properties[48]. This agent has an interesting history, having been developed in Japan where it has been marketed for almost 20 years for the treatment of asthma and cerebrovascular disorders. Two North American pharmaceutical companies, Avigen and MediciNova, have acquired the rights for development of ibudilast for the treatment of chronic, inflammatory neuropathic pain and multiple sclerosis, with Phase II clinical trials either underway or completed in the case of AV-411 and MN-166, respectively[48,49].

## ROLE OF MONTELUKAST AS THERAPY FOR DIVERSE INFLAMMATORY DISORDERS

Although registered primarily for use in asthma and/or allergic rhinitis, numerous trials, reviews, and reports have suggested that there may be additional disorders in which montelukast may be beneficial in therapy, and these are indicated in Table 1[50,51]. While in some of these other conditions, especially those associated with asthma, CysLTs may play a role in disease pathogenesis, and therefore the use of receptor antagonists in therapy may be completely predictable; in others, the $CysLT_1R$-independent activity may also contribute[6,7,8,9,13,36].

### Chronic Obstructive Pulmonary Disease (COPD)

The therapeutic potential of montelukast has been evaluated in COPD, a disorder in which leukotrienes may play a significant role[52]. However, targeting leukotrienes may not be the primary mechanism of benefit of $CysLT_1Rs$ in COPD, a condition in which the neutrophil is the predominant cell type recovered from the airway lumen[53,54]. PDE inhibitors, such as theophylline currently used in the treatment of this condition, appear to have bronchodilator, anti-inflammatory, and pulmonary vasodilator actions, probably attributable to increases in cAMP, resulting in reductions in cellular proliferation, smooth muscle relaxation, and decreased cellular inflammatory activity[53]. Alternatively, theophylline may increase histone deacetylase activity in alveolar macrophages from patients with COPD[55]. It is hardly surprising

**TABLE 1**
**CysLTR-Dependent and -Independent Anti-Inflammatory Activities of Montelukast, Together with the Disorders that may be Sensitive to Montelukast Consequent to Its Diverse Mechanisms of Action**

| Anti-Inflammatory Activities of Montelukast | Disorders |
|---|---|
| Primary | |
| Inhibition of CysLTRs. | Allergic rhinitis<br>Atopic asthma<br>Aspirin-induced bronchospasm |
| Secondary | |
| Inhibition of 5-lipoxygenase<br>Inhibition of PDEs<br>Suppression of HAT activity<br>Interference with P2Y receptor signaling<br>Inhibition of eosinophil adhesion to vascular endothelium | COPD, cystic fibrosis, viral bronchiolitis, idiopathic pulmonary fibrosis<br>Paranasal sinus disease, allergic fungal sinusitis, nasal polyposis, otitis media, allergic conjunctivitis<br>Chronic urticaria, atopic dermatitis, systemic mastocytosis<br>Atherosclerosis<br>Irritable bowel syndrome, pancreatitis, vulvovaginal candidiasis, interstitial cystitis<br>Sepsis<br>Immune reconstitution syndrome (IRIS)<br>Hepatic ischemia-reperfusion injury |

that new therapies for use in COPD have focused on the development of selective PDE4 inhibitors[53,56,57,58,59,60].

The short-term effects of montelukast in stable patients with moderate-to-severe COPD have been studied, with the end points being the dyspnea score, arterial blood gases, lung function tests, and quality of life (QoL) scores[61]. This was a prospective, randomized, single-blind controlled study of 117 patients with COPD evaluated over a 2-month period. Significant increases in vital capacity, FVC, $FEV_1$, visual analog scores (VAS), and $PaO_2$ were noted at 2 months ($p < 0.05$) and there was a significant improvement in QoL scores ($p < 0.05$). Sputum samples were obtained in 24 of the COPD cases, and in the montelukast group, a decrease in neutrophilic activity was evident (n = 13; $p = 0.059$). The authors concluded that leukotriene antagonists should be considered for use in COPD patients when additional anti-inflammatory activity is needed.

In a retrospective study, the long-term effects of montelukast on the control of COPD were investigated in a small cohort of patients with moderate-to-severe COPD[62]. The duration of follow-up was 23.6 ± 7.3 months. There was a significant improvement in reported shortness of breath, sputum production, wheezing, and nocturnal symptoms during the observation period ($p < 0.05$), as well as a significant reduction in the use of oral or inhaled corticosteroids, inhaled bronchodilators, and supplemental oxygen ($p < 0.05$). There was also a significant reduction in visits to the emergency department, number of hospitalizations, and duration of hospitalization for acute exacerbations of COPD ($p < 0.05$). No significant changes were noted with regard to $FEV_1$, $FEV_1$/FVC ratio, or peak flow. No side effects were reported and no patients discontinued medications. The authors concluded that long-term use of montelukast was safe and improved control in moderate-to-severe COPD. More recently, a small study of patients with stable COPD, in whom montelukast was added, was undertaken over a 12-month period[63]. This study documented a decrease in serum levels of $LTB_4$, IL-8, and TNF, together with a reduction in dyspnea and sputum production, a decrease in the number of outpatient clinic visits, hospitalizations, and duration of hospitalization following initiation of montelukast.

## Cystic Fibrosis (CF)

In CF, the inflammatory mediators and mechanisms involved are such that they could be considered potential targets for both $LTB_4$ receptor and $CysLT_1R$ antagonism[64], with several studies having documented the benefit of montelukast[65,66]. In a study of patients with mild CF, beneficial effects were seen on eosinophilic inflammation, but no improvement was documented in clinical symptom scores, IL-8 levels, or lung function studies over the short period of montelukast administration (3 weeks)[65]. However, in a 20-week study of patients with moderate CF, significant improvements in cough and wheezing scale scores and lung functions ($FEV_1$, PEF, FEF 25–75%) were documented, as well as decreases in eosinophilic inflammation and IL-8 levels[66].

## Viral Bronchiolitis

Following respiratory syncitial virus (RSV) bronchiolitis, infants often develop reactive airways disease. Although it is thought that this is associated with release of CysLTs, other pathways may be involved. In one study, 133 infants, aged 3–36 months, were enrolled in a double-blind parallel comparison of 5-mg montelukast or placebo for 28 days, starting within 7 days of symptom onset[67]. Infants on montelukast were free of symptoms on 22% of days and nights compared to 4% in the placebo group ($p = 0.015$), daytime cough was significantly reduced in the active group ($p = 0.04$), and exacerbations were significantly delayed in the montelukast group in comparison to placebo ($p < 0.05$). A more recent experimental study in mice confirmed the benefit of montelukast in attenuating the RSV-induced airway hyper-responsiveness and inflammation[68]. In addition, a recent review has described the increasing evidence for use of $CysLT_1R$ antagonists in virus-induced wheezing[69].

## Sepsis

A number of studies, primarily involving experimental animal models, have suggested that PDE inhibitors or dibutyryl cAMP may be of value in the treatment of sepsis and endoxin-induced shock. These agents may attenuate sepsis-associated organ dysfunction involving the heart[70,71], lungs[72,73], liver[74], skeletal muscle[75], and kidneys[76,77,78]. Although clinical studies in humans are lacking, recent data, albeit somewhat preliminary, have indicated a potential role for montelukast in the anti-inflammatory therapy of sepsis. First, in models of experimental infection, montelukast inhibits bacterial penetration of human brain microvascular endothelial cells, attenuating invasion of the brain and counteracting development of *Escherichia coli* meningitis[79]. Second, montelukast has been shown to ameliorate sepsis-induced hepatic and ileal injury in a rat model by protecting the animals from neutrophil-mediated oxidative injury[80].

The clinical and experimental settings in which the $CysLT_1R$-independent activities of montelukast may contribute to the therapeutic efficacy of this agent are listed in Table 1. It is currently unknown whether these secondary anti-inflammatory activities are limited to montelukast or represent a class effect. Furthermore, if operative in the therapeutic setting, they may complement the primary activity (antagonism of CysLTRs) of this agent.

## CONCLUSION

Montelukast has been reported to possess secondary anti-inflammatory properties, apparently unrelated to conventional antagonism of $CysLT_1Rs$. These novel activities enable montelukast to target eosinophils, monocytes, and, in particular, the corticosteroid-insensitive neutrophil, suggesting that this agent may have a broader spectrum of anti-inflammatory activities than originally thought.
